# Expression of the Rice Arginase Gene *OsARG* in Cotton Influences the Morphology and Nitrogen Transition of Seedlings

**DOI:** 10.1371/journal.pone.0141530

**Published:** 2015-11-03

**Authors:** Zhigang Meng, Zhaohong Meng, Rui Zhang, Chengzhen Liang, Jianmin Wan, Yanling Wang, Honghong Zhai, Sandui Guo

**Affiliations:** 1 Biotechnology Research Institute, Chinese Academy of Agricultural Sciences, Beijing, China; 2 Institute of Crop Science, Chinese Academy of Agricultural Sciences, Beijing, China; 3 School of Life Science, Anhui Agricultural University, Anhui, China; National Key Laboratory of Crop Genetic Improvement, CHINA

## Abstract

Arginase is the only enzyme capable of producing urea in plants. This enzyme also contributes to many important biological functions during plant growth and development, such as seed development, root development and plant nitrogen using. The unique rice arginase gene *OsARG* is known to affect nitrogen use efficiency and is also associated with higher yields in rice. In this study, we transformed *OsARG* into upland cotton R18 by *Agrobacterium*-mediated genetic transformation and analyzed the function of *OsARG* in transgenic cotton. Two independent *OsARG* expression transgenic cotton lines, ARG-26 and ARG-38, were obtained via transformation. Southern blot analysis indicated that two copies and one copy of the *OsARG* gene were integrated into the ARG-26 and ARG-38 genomes, respectively. Enzyme activity and RNA transcription analysis revealed that the *OsARG* gene is highly expressed in cotton. The nitric oxide content and the morphology of ARG-26 and ARG-38 seedlings were both affected by expression of the *OsARG* gene. Field experiments indicated that the polyamine and nitrogen content increased by more than two-fold in the T3 generation plants of the transgenic cotton lines ARG-26-2, ARG-26-7, ARG-38-8, and ARG-38-11, as compared with the control plants. After harvesting cotton fibers grown in field conditions, we analyzed the quality of fiber and found that the fiber length was increased in the transgenic lines. The average cotton fiber length for all of the transgenic cotton lines was two millimeters longer than the fibers of the control plants; the average cotton fiber lengths were 31.94 mm, 32.00 mm, 32.68 mm and 32.84 mm in the ARG-26ARG-26-2, ARG-26-7, ARG-38-8 and ARG-38-11 lines, respectively, but the average fiber length of the control plants was 29.36mm. Our results indicate that the *OsARG* gene could potentially be used to improve cotton fiber length traits.

## Introduction

The application of nitrogen (N) is an important practice in cotton production. The quantity of nitrogen fertilizer applied and the nitrogen use efficiency of cotton plants both strongly influence cotton yield and fiber quality [1, 2], and these factors are also known to influence plant defense responses to biotic and abiotic stress [3]. Efforts to improve nitrogen utilization efficiency aimed at improving cotton yield and quality have been of particular significance for cotton production in nitrogen-limited conditions. Plants mainly acquire nitrogen via three steps: uptake, assimilation, and remobilization. Plant nitrogen uptake and assimilation determine external nitrogen use efficiency from soil, while nitrogen remobilization determines the in planta efficiency of nitrogen storage and reutilization. Nitrogen remobilization is an important factor influencing plant nitrogen use efficiency; high nitrogen remobilization efficiency can cause extra organic state nitrogen to be restored and reused in plant metabolism, a situation that prevents the conversion of this nitrogen into nitric oxide (NO) and other inorganic nitrogen forms that would be wasted. Examples of nitrogen remobilization include transamination metabolism processes that synthesize new amino acids and protein to achieve reuse of organic nitrogen and the synthesis of some transferable amino acids that are used in nitrogen translocation between old leaves and developing or photosynthetically-active leaves or seeds and other reproductive organs [4, 5]. In most plant species, nitrogen remobilization is mainly executed via arginine (Arg) metabolism. Arg metabolism has an important significance for processes including seed development, germination, and seedling growth and development. Arg is a primary storage form of nitrogen in seeds, often accounting for 50% of the content of nitrogen storage. After germination, Arg content can account for 90% of the soluble nitrogen in the tender tissues of seedlings [6, 7, 8].

Compared to amino acids generally, Arg, which contains a guanidine and an amino group, has a particularly high nitrogen content. Arg is also known to be an important nitrogen storage molecule and an important transport form for nitrogen translocation [9, 10]. In plant Arg metabolism, Arg is a precursor for the biosynthesis of some important functional molecules such as amino acids, polyamines, agmatine, and NO. Polyamines and agmatine are associated with plant resistance to environmental adversity. NO is involved in signal transduction in plants [11, 12]. Arginase can transform endogenous nitrogen into organic nitrogen to be reused in plant by hydrolyzing Arg to produce urea and ornithine (Orn). Urea can be utilized in the urea cycle to reuse nitrogen for the synthesis of amino acids and proteins; ornithine can be used as a precursor for the synthesis of both polyamines and proteins [13, 14, 15]. Arginase is involved in the production of many important molecules, and the biological functions of these molecules are receiving increasing amounts of research attention in a variety of plants [11, 16, 17, 18].

Ma *et al*. [16] found that, in the rice mutant nglf-1 for the arginase gene (*OsARG*), plant height, panicle number, seed size, and seed setting rate were all decreased compared to wild type plants, The seed setting rate phenotype was particularly pronounced in the mutant, with a more than 80% decreased compared to the control. Gene expression profile analysis and transgenic expression studies in rice indicated that *OsARG* was associated with nitrogen use efficiency and with seed development. The number of seeds per plant in transgenic plant was significantly increased, by more than 20%, compared with control plants under nitrogen-limiting conditions.

In this study, we cloned the rice arginase gene *OsARG* (CDS: HM369061) and constructed a plant expression vector. Transgenic cotton was created by genetically transforming cotton using *Agrobacterium tumefaciens*. We analyzed the seedling morphology and fiber length, as well as the amino acid, polyamine, and nitrogen content, of transgenic cotton plants to evaluate the function of *OsARG* when expressed in cotton. The results of this study provide a theoretical basis for understanding the role of this gene in nitrogen use efficiency and suggest this gene as a candidate for use in breeding efforts aimed at improving cotton production.

## Materials and Methods

### Biological materials

Seeds of the cotton cultivar Coker 312 (*Gossypiumhirsutum* L.) were obtained from the Institute of Cotton Research Shanxi Academy of Agricultural Sciences, China. Seeds were delinted with 98% H2SO4. The *Agrobacterium tumefaciens* strain AGL-1was used to transform the genes into cotton.

### Growth conditions for cotton plants

DNA, RNA, and proteins were isolated from cotton plants grown in a growth chamber on 1/2 MS medium with varying nitrogen content (1/2 MS medium, 1/2 MS medium with 4×NH_4_NO_3_, and 1/2 MS medium lacking NH_4_NO_3_ and KNO_3_). The light period was set from 7:00 h to 22:00 h (15 h light) at 28°C; the temperature during the dark period was 25°C. Samples for nitrogen and fiber analysis were collected from plants grown under standard field conditions. Samples were collected from three independent field areas, and three replicate plants were sampled for each line from each of the areas.

### Construction of the expression vector

The transit peptide from *OsARG* CDS (HM369061) was deleted based on sequence information from UniProtKB (B8AU84). The remaining sequence was fused with the mitochondrial transit peptide from the *Arabidopsis thaliana* aldehyde dehydrogenase gene (ALDH2B7) via PCR. The fusion gene, under the control of the CaMv35S promoter and the NOS terminator, was introduced into the pBI121 vector to create the transformation plasmid. The PCR primers used were ARG_FP and ARG_RP (Table 1).

**Table 1:** Concentrations of amino acids in the leaves of control and transgenic cotton plants.

Amino acid	Control	ARG-26-2	ARG-26-7	ARG-38-8	ARG-38-11
Ala	61.73±0.31	48.26±0.31	48.26±0.33	56.12±0.3	54.99±0.29
Asp	54.85±0.13	66.12±0.12	48.08±0.15	48.84±0.14	53.34±0.17
Asn	1161.24±1.75	1490.54±1.58	1489.78±1.8	1489.78±1.55	1491.29±1.61
Cys	21.45±0.41	20.63±0.56	18.15±0.49	19.8±0.52	21.45±0.38
Gly	54.59±0.11	53.26±0.18	49.27±0.13	50.6±0.15	55.93±0.15
Gln	77.29±0.29	87.55±0.32	88.92±0.28	87.55±0.31	88.24±0.31
His	11.60±0.08	12.24±0.09	9.66±0.06	10.31±0.08	10.95±0.05
Ile	26.68±0.13	25.91±0.12	25.15±0.14	25.91±0.19	28.20±0.12
Leu	56.40±0.32	51.83±0.35	51.83±0.33	52.59±0.32	55.64±0.33
Met	10.05±0.03	10.05±0.08	8.71±0.07	6.7±0.05	10.72±0.09
Ser	34.25±0.27	28.54±0.22	29.5±0.21	30.45±0.25	29.50±0.23
Thr	32.75±0.25	31.91±0.23	31.07±0.26	30.23±0.19	31.07±0.21
Val	43.55±0.26	40.99±0.19	38.43±0.2	40.99±0.18	39.28±0.23
Arg	32.15±0.33	20.54±0.32*	20.68±0.34*	20.68±0.30*	20.96±0.31*
Glu	61.18±0.11	47.00±0.13*	47.38±0.15*	47.78±0.18*	47.78±0.11*
Lys	41.04±0.20	28.99±0.21*	26.25±0.20*	27.62±0.17*	28.04±0.19*
Pro	26.06±0.24	12.46±0.25*	12.46±0.25*	12.46±0.29*	12.43±0.30*
Orn	17.97±0.15	32.28±0.12◆	31.92±0.11◆	32.64±0.08◆	32.28±0.13◆
Phe	23.00±0.36	41.18±0.44◆	41.06±0.42◆	41.24±0.41◆	41.27±0.48◆
Trp	13.22±0.05	27.63±0.08◆	27.61±0.03◆	27.55±0.08◆	27.56±0.07◆
Tyr	24.28±0.39	41.46±0.41◆	41.41±0.39◆	41.59±0.32◆	41.60±0.40◆

Values are the means ± SD (μmol/g DW). ‘Control’ refers to non-transgenic control plants.

‘*’ indicates that the amino acid content of the transgenic line was significantly decreased compared with that of control.

‘◆’ indicates that the amino acid content of the transgenic line was significantly increased compared with that of control.

‘Control’ refers to non-transgenic wild type plants. P ≤ 0.05.

### Genetic transformation and screening of cotton transformants

The method of cotton transformation and the tissue culture conditions used in this study were as previously described in Meng *et al* [19]. Hypocotyls were excised from 5- to 6-day-old seedlings and used as explants for callus induction. All offspring from each generation of transgenic cotton were screened via PCR and for the presence of the kanamycin resistance gene *Npt*II, and then bred via self-fertilization. The PCR primer pair *OsARG*-F and *OsARG*-R (S1 Table) was used to amplify a 966 bp fragment of the *OsARG* gene. The PCR cycles were as follows: pre-denaturation at 94°C for 5 min; 30 cycles of amplification (94°C for 30 s, 58°C for 30 s, 72°C for 30 s); followed by 5 min at 72°C. Homozygous non-transgenic plants separated from the T2-generation transgenic plants were bred as a control (Control) and used throughout this study.

### DNA extraction, RNA extraction and cDNA synthesis

Fresh cotton leaves were used to extract genomic DNA using the cetyltrimethyl ammonium bromide (CTAB) method [20]. Total RNA from leaves was extracted using an EASYspin RNA extraction kit (Yuan Ping Hao Bio Co., Ltd., China; Cat. No. HF109-02) according to the manufacturer’s instructions. RNA and DNA quality and concentrations were evaluated using spectrophotometric analysis and agarose gel electrophoresis with ethidium bromide staining. First strand cDNA was synthesized using a ReverTra Ace-α- cDNA synthesis kit (TOYOBO Co., Ltd., Japan; Cat. No.FSK-100), according to the manufacturer’s instructions.

### DNA Southern blot analysis

Southern blots were performed using an [α-^32^P] dCTP DNA labeling probe and standard detection methods. Genomic DNA (10μg/sample) was digested overnight at 37°C using *Eco*R I and *Hind* III.

After the digested DNA fragments were separated by gel electrophoresis (0.8% agarose gel), the DNA fragments were transferred to a Amersham HybondTM-XL nylon membrane (GE Healthcare Limited, USA; Cat. No.RPN303S) in 10× SSC buffer (1.5 mol/L NaCl and 0.15 mol/L sodium citrate, pH 7.0) for 12 hours at room temperature. The DNA was then fixed to the nylon membrane. The probes were labeled using a Prime-a-Gene® Labeling System (Promega Co., USA; Cat. No.U1100) according to the manufacturer’s instructions. The templates for [α-^32^P] dCTP probe labeling were products amplified using the primer pair OsARG-F/OsARG-R. The prehybridization and hybridization processes were carried out in 20 mL Church buffer (Na_2_HPO_4_ 0.5 mol/L pH7.2, EDTA 0.001 mol/L pH8.0, BSA 1%, SDS 7%) and 20μgssDNA (1μg /mL), respectively. Prehybridization and hybridization took place in a hybridization oven at 68°C for 4 hours and 10 hours, respectively. After hybridization, the nylon membrane was washed once in 0.5× SSC with 0.1% SDS for 2 minutes at room temperature and washed twice in 0.5× SSC with 0.1% SDS for 15 minutes at 68°C. The nylon membrane was exposed to FujiFilm for 24 hours using Fisher Biotech L-Plus intensifying screens. Hybridization bands were visualized on FujiFilm using a Typhoon FLA 7000 scanner (GE Healthcare Limited, USA).

### Quantitative real-time PCR

Quantitative real-time PCR was performed with SYBR® Green Real-time PCR Master Mix (TOYOBO Co., Ltd.; Cat. No. QPK-201) as described in the manufacturer’s instructions. Total RNA from the leaves and the cDNA analyzed by real-time PCR were prepared as described above. The reference gene used for normalization of the real-time PCR reactions was the cotton elongation factor 1α gene (*GhEF1α*).The primers for *GhEF1α* were *EF1α*_F and *EF1α*_R (S1 Table). The specific primers for *OsARG* were *OsARG*_QPCR_FP and *OsARG*_QPCR_RP (S1 Table). The real-time PCR program included the following steps: pre-denaturation at 98°C for 30 s; amplification for 40 cycles (98°C for 10 s, 60°C for 30 s, 72°C for 30 s, then the plate was held at 78°C for 2 s so fluorescence data could be read from the plate); then elongation at 72°C for 5 min. Amplification product melting curve analyses were performed to confirm the specificity of the quantitative real-time PCR reactions. The temperatures were set from 55°C to 95°C (with a 2 s hold per 0.5°C increase). The threshold value was 0.5, and the threshold cycles (CT) were calculated using the Chromo4 Real-Time PCR System (Bio-Rad Laboratories, Inc. USA) software. The calculation method for relative quantification reported by Pfaffl [21] was used.

### Arginase enzyme activity analysis

Leaves (approximately 0.1 g) were collected from one month-old *OsARG* transgenic cotton plants and ground with 500 μL of ice-cold extraction solution (1 mM MnSO_4_, 50 mM Tris, pH 9.0) to extract total protein. The solution was centrifuged at 18,000 g for 20 min at 4°C. After removal of the supernatant, arginase activity was measured using a QuantiChrom™ Arginase Assay Kit (BioAssay Systems, USA; Cat. No. DARG-200) according to the manufacturer’s instructions. The protein concentrations of the samples were determined using a BCA protein assay kit (Bio-Rad Laboratory Inc.;Cat. No.500-0116) with BSA used as the standard.

### Nitric oxide content analysis

Seedlings from T1 generation of ARG-26 and ARG-38 cotton were cultured in 1/2 MS medium with varying nitrogen content. After the plant height, root length, lateral root number, and root length were analyzed, the aerial portions and the roots of 15cotton plants from each line were combined as a single sample and ground. The samples were used to analyze the NO content using a total nitric oxide content analysis kit (Beyotime Institute of Biotechnology, China; Cat. No. S0023).

### Analysis of amino acid, polyamine, and nitrogen content per unit weight of cotton leaf

Penultimate leaves from 15 independent cotton plants from each line were collected when the bud appeared and then combined into a single sample and ground to a powder in liquid nitrogen. The accumulation of amino acids [22], polyamines [23], and nitrogen content [24] per unit leaf weight were measured.

### Cotton fiber length analysis

Seed cotton from each line was harvested in the field. The fiber lengths were measured using a cotton fiber photoelectric length measuring instrument (Chang Zhou Tian Xiang Textiles Instruments Factory Co., Ltd, China, Y146-3B). The average fiber length of each transgenic cotton line and the control was calculated.

## Results

### Construction of the expression vector

**Fig 1:**
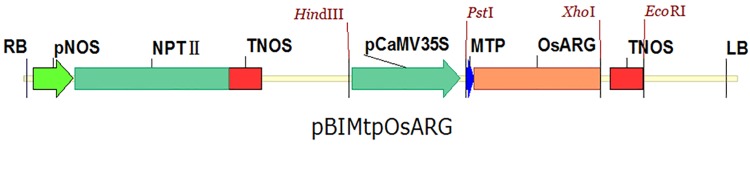
Map of the plant expression vector.

Rice arginase has a transit peptide and is known to be localized to mitochondria. Given the existence of certain differences in subcellular localization mechanisms between monocots and dicots, we hypothesized that the rice arginase gene could be expressed in cotton and targeted into mitochondria if a rice arginase construct replacing its native transit peptide with a dicot mitochondrial transit peptide was introduced into cotton transgenically. A new ORF was constructed that replaced the transit peptide nucleotide sequence (60bp, 20AA) of the *OsARG* CDS (HM369061, 1023bp, 340AA) with the mitochondrion transit peptide (Mtp) nucleotide sequence (63 bp, 21AA) of the *Arabidopsis thaliana* aldehyde dehydrogenase gene (AtALDH, ALDH2B7).The new *OsARG* ORF was inserted into the PUC19 plasmid using the *Pst*I and *Xho*I sites. Promoter and terminator sequences were inserted into PUC19 *OsARG* via enzyme digestion. The CaMv35S promoter was digested using *Pst*I and *Hind*III, and the NOS terminator was digested using *Pst*I and *Eco*RI. The transformed plasmid pBIMtp*OsARG* was constructed by inserting CaMv35S::*OsARG*:: NOS into pBI121 using *Hind*III and *Eco*RI (Fig 1).

### PCR and Southern blot analysis of transgenic cotton

**Fig 2:**
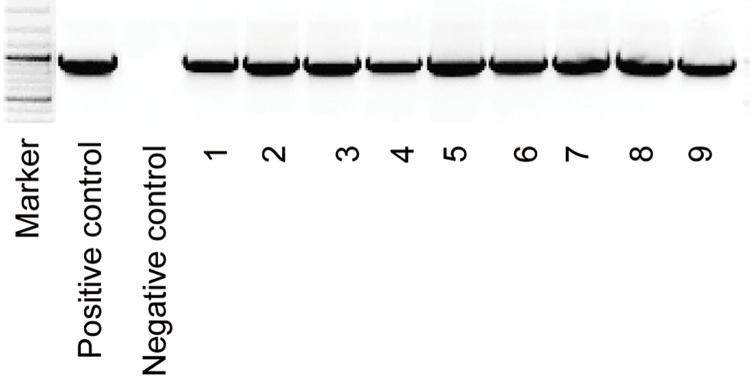
PCR identification of the *OsARG* gene in transgenic cotton. The under labels indicate the following samples: M, marker. 1, positive control. 2, negative control. 3–6, PCR analysis of four independent cotton plants containing ARG-26 genomic DNA. 7–10, PCR analysis of four independent cotton plants containing the ARG-38 genome DNA.

**Fig 3:**
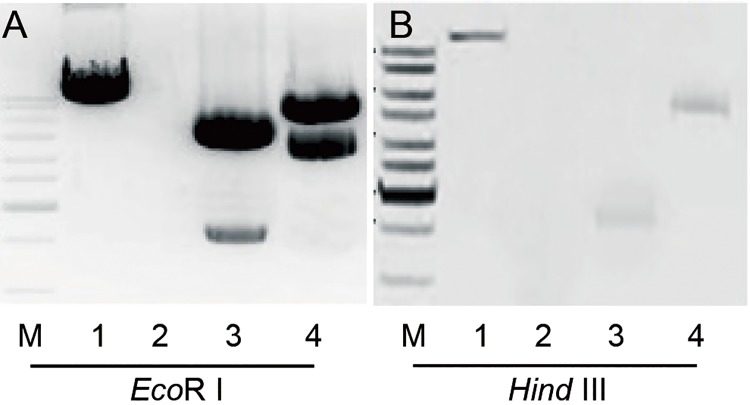
Southern blot analysis of *OsARG* gene copy number in the genomes of transgenic cotton plants. The probe was labeled with the radioactive isotope [α-^32^P] dCTP. (A) Two copies of *OsARG* were integrated into the ARG-26 genome. (B) One copy of *OsARG* was integrated into the ARG-38 genome. The under labels indicate the following samples: M, marker. 1, positive control. 2, negative control. 3, genomic DNA digested using *Eco*RI. 4, genomic DNA digested using *Hind*III.

Two transgenic cotton plants were obtained by *Agrobacterium*-mediated genetic transformation; they each exhibited normal growth characteristics, designated as ARG-26 and ARG-38. The offspring of these plants were screened using PCR amplification of the *OSARG* gene in kanamycin resistant cotton plants; the size of the PCR product for the positive plants was shown as Fig 2. Southern blot analysis was used to investigate the number of *OsARG* copies integrated into the genomes of the T1generation cotton plants. Genomic DNA from transformed cotton plants was digested using *Eco*RI and *Hind*III. The probe length was 966 bp No *Eco*RI or *Hind*III restriction sites were present in the probe region. The hybridization results indicated that there were two strong bands detected in the *Eco*RI and *Hind*III lanes of the ARG-26 genomic DNA (Fig 3A).One band was detected in the *Eco*RI and *Hind*III lanes of the ARG-38 genomic DNA (Fig 3B). These results suggested that two copies of the *OsARG* gene were integrated into the ARG-26 genome, and that one copy was integrated into the ARG-38 genome. T3 generation homozygous offspring originating from ARG-26 and ARG-38 were bred by self-pollination.

### Analysis of *OsARG* expression using quantitative PCR

**Fig 4:**
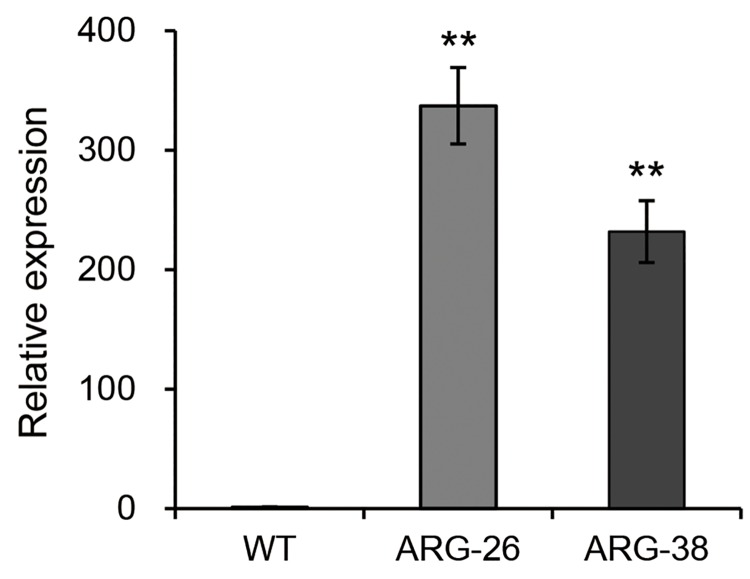
Quantitative PCR analysis of OsArg expression in the leaves of ARG-26 and ARG-38 transgenic cotton. The *OsARG* gene was detected in ARG-26 and ARG-38 leaves but not in non-transgenic control leaves. ‘WT’ refers to non-transgenic control plants of wild type cotton. **: P<0.01

Quantitative PCR was performed to determine whether mRNA transcripts of the *OsARG* gene were present in the transgenic cotton plants. The relative average expression values for the gene in the ARG-26 and ARG-38 plants were significantly higher than that measured in the control plants (homozygous plants bred from separated non-transgenic plants in the T2 generation).The results indicated that the *OsARG* gene was expressed at high levels (Fig 4).

### Arginase activity analysis

**Fig 5:**
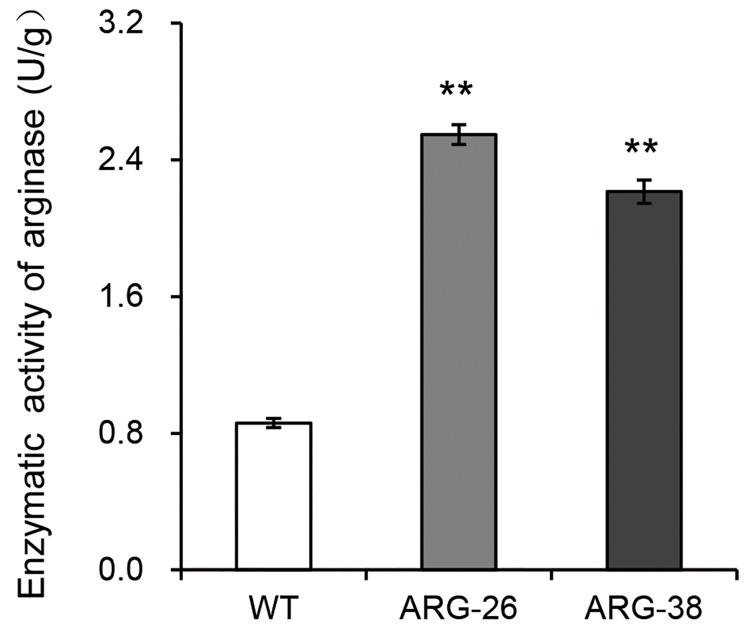
Arginase enzyme activity in ARG-26 and ARG-38 transgenic cotton leaves. Compared with arginase activity of non-transgenic control plants, expression of OsARG increase the total arginase activity in ARG-26 and ARG-38. ‘WT’ refers to non-transgenic control plants of wild type cotton. **: P<0.01.

We conducted enzyme assays to determine whether the expressed *OsARG* protein was able to perform arginase enzyme activity in transgenic cotton. Total protein from one unit of fresh leaf weight of ARG-26 and ARG-38 were used to hydrolyze Arg to produce urea. And the content of produced urea by hydrolyzation of 1μmol Arg with the total protein from one unit fresh leaf weight of ARG-26 and ARG-38 designated as arginase enzyme activity. The results indicated that total arginase enzyme activity in ARG-26 and ARG-38 plants were significantly higher than in the control plants (Fig 5). The average enzyme activity in the leaves of ARG-26 seedlings was 2.55U/g (fresh leaf weight); this value was 2.9-fold greater than the enzyme activity measured in control plants. The average enzyme activity in the leaves of ARG-38 seedlings was 2.21U/g (fresh leaf weight), which was 2.5-fold greater than that in control plants.

### The effect of *OsARG* expression on the morphology of cotton seedlings grown with differing levels of nitrogen

In the process of seed germination and seedling growth, the main nitrogen nutrient source is known to be storage proteins in cotyledons. The remobilization of storage proteins occurs mainly via Arg metabolism. Increased arginase activity will degrade more Arg, producing urea and ornithine to accelerate remobilization of storage nitrogen from plants and use of absorbed nitrogen from soli [6]. To determine whether the *OsARG* gene affects cotton seedling development, homozygous seedlings from the T3 generation of ARG-26 and ARG-38 cotton were cultured in 1/2 MS medium with varying nitrogen content. The values for average seedling height (growth above the solid medium), the average taproot length, the average number of lateral roots, and the average length of lateral roots were measured at 14 days after germination (DAG).

**Fig 6:**
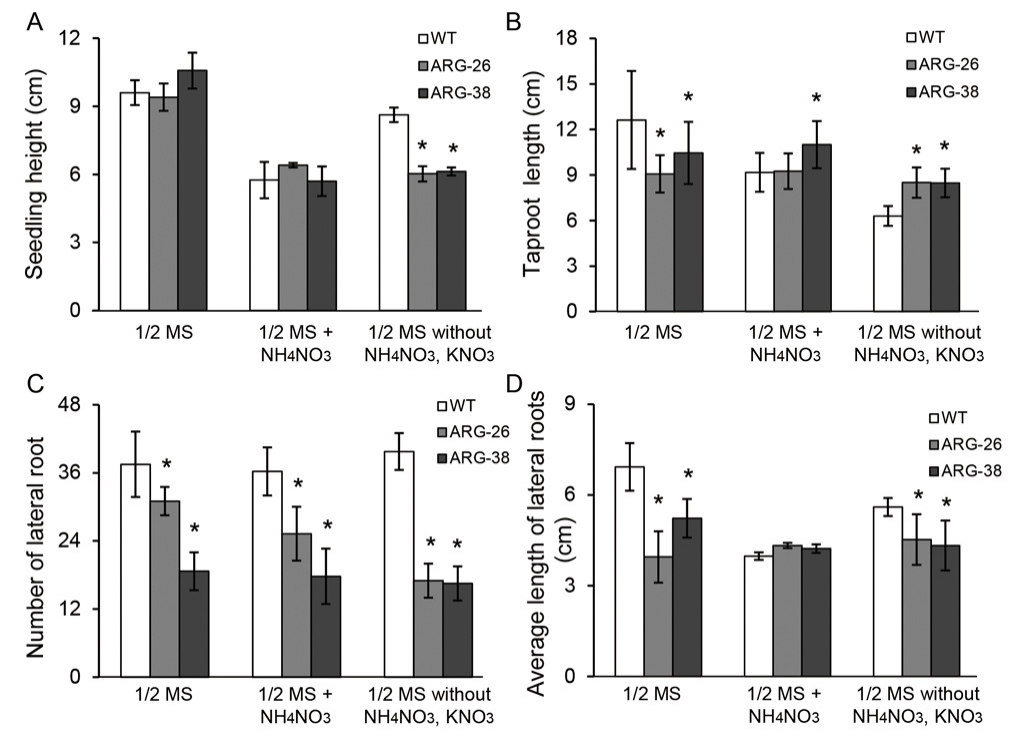
Morphology analyses of transgenic cotton seedlings grown on 1/2 MS medium with varying nitrogen content. T4-generation ARG-26 and ARG-38 cotton seedling were cultured on three kinds of growth media:1/2 MS medium, 1/2 MS medium with 4×NH_4_NO_3_, and 1/2 MS medium lacking nitrogen. Due to the expression of the *OsARG* gene in cotton, the seedling height, taproot length, lateral root number, and lateral root length of ARG-26 and ARG-38 plants were changed compared with those of non-transgenic control cotton plants. Both of the transgenic seedling heights were same or increased on 1/2 MS medium and 1/2 MS medium with 4×NH_4_NO_3_, but decreased on 1/2 MS medium lacking nitrogen. Both of the transgenic seedling taproot lengths were same or increased on 1/2 MS medium with 4×NH_4_NO_3_ and 1/2 MS medium lacking nitrogen, but decreased on 1/2 MS medium. Both of the transgenic seedling lateral root numbers were decreased on 1/2 MS medium,1/2 MS medium with 4×NH_4_NO_3_ and 1/2 MS medium lacking nitrogen. Both of the transgenic seedling lateral root lengths were little increased on 1/2 MS medium with 4×NH_4_NO_3_, but decreased on 1/2 MS medium and 1/2 MS medium lacking nitrogen. (A) the seedling height of transgenic cotton was analyzed for plants grown on three kinds of growth media. (B) taproot length was analyzed of transgenic cotton was analyzed for plants grown on three kinds of growth media. (C) and (D) the average number and the average length of lateral roots were analyzed for plants grown on three kinds of growth media for plants grown on three kind growth media. ‘WT’ refers to non-transgenic control plants of wild type cotton. *: P<0.05.

Compared with control plants, the average height of ARG-26 and ARG-38 seedlings were inversely correlated with taproot length (Fig 6A and 6B).When ARG-26 and ARG-38 seedlings were grown on 1/2 MS medium, the average height of the transgenic seedlings were greater than or similar to the height of the control seedlings; the average taproot lengths of the transgenic seedlings were shorter than those measured for control seedlings. However, the average seedling height and the average taproot length of ARG-38 were each greater than those of ARG-26. When ARG-26 and ARG-38 seedlings were grown on 1/2 MS medium with 4×NH_4_NO_3_, the average seedling height of ARG-26 was greater than that of the control; the average taproot length of ARG-26 was not significantly different from that of the control. The average height and the average taproot length of the ARG-38 seedlings were inversely correlated with the corresponding measurements for the ARG-26 seedlings. However, when ARG-26 and ARG-38 seedlings were grown on 1/2 MS medium without NH_4_NO_3_ and KNO_3_, the average heights of the ARG-26 and ARG-38 seedlings were shorter than the control seedlings; under these conditions, the average taproot lengths of ARG-26 and ARG-38 were longer than that of the control. No differences in the average height or average taproot length were observed between the ARG-26 and ARG-38 seedlings.

The average number of lateral roots and the average length of lateral roots indicated that *OsARG* expression affected lateral root development to a statistically significant extent (Fig 6C and 6D). When grown on 1/2 MS medium with varying nitrogen content, the average numbers of lateral roots ofARG-26 and ARG-38 were decreased compared to the control. The average number of lateral roots for ARG-26 plants was greater than that for ARG-38 plants in the varying media. The removal of NH_4_NO_3_ and KNO_3_ from the 1/2 MS medium significantly reduced the development of lateral roots in ARG-26 and ARG-38 seedlings. *OsARG* expression reduced the length of lateral roots when transgenic seedlings were grown on 1/2 MS medium or 1/2 MS medium without NH_4_NO_3_ and KNO_3_. However, the average length of lateral roots in transgenic seedlings was slightly increased compared with that of the control when the plants were grown on 1/2 MS medium with 4×NH_4_NO_3_.

### Nitric oxide content in transgenic cotton seedlings

**Fig 7:**
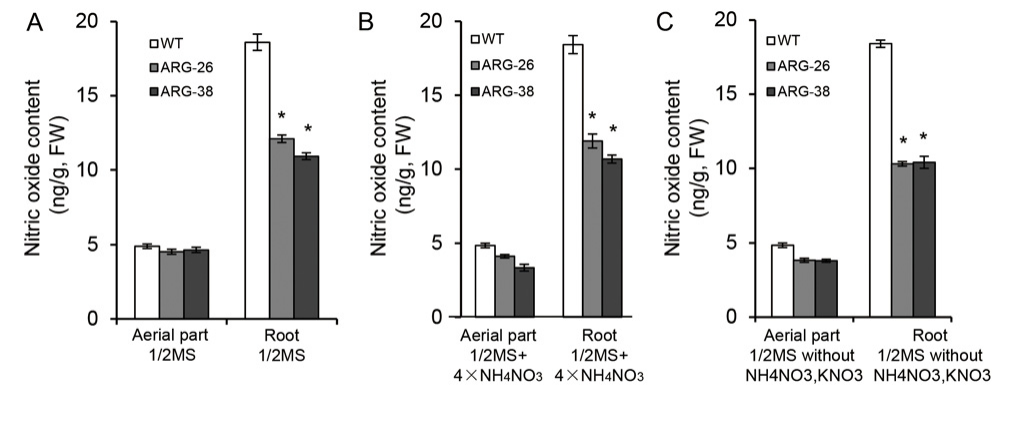
Nitric oxide content analysis in the aerial parts and in the roots of ARG-26 and ARG-38 seedlings grown on 1/2 MS medium, 1/2 MS medium with 4×NH4NO3, and 1/2 MS medium lacking nitrogen. Compared to non-transgenic control plants, the nitric oxide content decreased in the aerial parts and in the roots of ARG-26 and ARG-38 seedlings on all of the various 1/2 MS media compositions. no differences were observed for the aerial parts of the plants. (A) the nitric oxide content in seedlings of transgenic cotton grown on 1/2 MS medium. (B) the nitric oxide content in transgenic cotton seedlings grown on 1/2 MS medium with 4× NH_4_NO_3_. (C) the nitric oxide content in transgenic cotton seedlings grown on 1/2 MS medium lacking NH_4_NO3 and KNO3. ‘FW’ refers to fresh leaf weight. ‘WT’ refers to non-transgenic control plants of wild type cotton. *: P<0.05.

Taproot and root hair development are regulated by nitric oxide (NO) content in roots. Arginase can influence NO synthesis and thus regulate plant root development by controlling the Arg content [13]. The effect of *OsARG* expression on the accumulation of nitric oxide in cotton seedlings was evaluated in our study. ARG-26 and ARG-38 seedlings were grown on various formulations of 1/2 MS media and morphological characteristics were observed at 14DAG. The seedlings were then collected to analyze the average NO content in the aerial parts and roots of the plants. For 15 selected plants, the aerial tissue and the roots were combined and measured for nitric oxide content. The results indicated that there were not obvious differences in the nitric oxide content in the aerial portions of the two transgenic cotton lines. However, the nitric oxide content in ARG-26 and ARG-38were significantly decreased compared to that of the control under 1/2 MS media with varying nitrogen content (Fig 7).

### The influence of *OsARG* expression on the amino acid content and accumulation of polyamines in cotton grown in the field

It is known that increased arginase activity can accelerate the metabolism of Arg and change the amino acid composition and polyamine content in plants. To explore the impact of *OsARG* expression on the nitrogen content of cotton grown in a normal field environment, four homozygous T3 generation lines expressing *OsARG* (ARG-26-2, ARG-26-7, ARG-38-8 and ARG-38-11) were selected for further analysis. Homozygous plants were also bred from separate, non-transgenic plants in the T2 generation and used as a control. Penultimate leaves of 15 independent cotton plants were selected from each line when the first bud appeared; these leaves were mixed together as a single and ground. The accumulation of amino acids and polyamines in the leaves was analyzed per unit weight of fresh leaf.

Amino acid analysis revealed that increased arginase activity led to decreased arginine content in all of the *OsARG* transgenic cotton lines (Table 1). Interestingly, expression of arginase was accompanied by increases in L-ornithine, L-phenylalanine, L-tryptophan, and L-tyrosine contents in all four the transgenic lines, as compared to the control plants. And decreases in the content of L-glutamic acid, L-lysine, and L-proline in all four lines were also observed. The content of the other amino acids were altered in the transgenic lines; however, no significant differences were observed in the levels of these compounds between the transgenic lines and the control plants.

**Fig 8:**
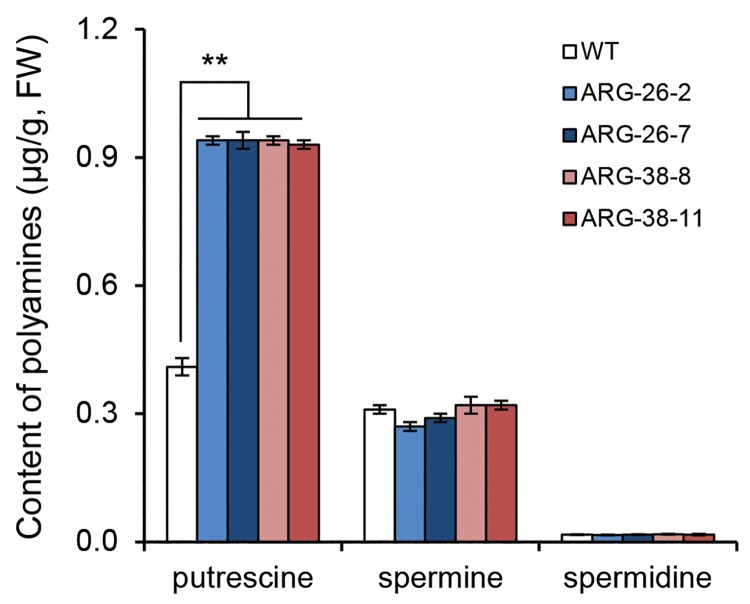
Comparison of the polyamine content in transgenic and control cotton plants grown under field conditions. The polyamine content here refers to the content of putrescine, spermine, and spermidine. All data in this figure are displayed as the mean values for putrescine, spermine, and spermidine content in the WT, ARG-26-2, ARG-26-7, ARG-38-8, and ARG-38-11 plants. The spermine and spermidine content in transgenic cotton plants were both imilar to those of the WT plants. The putrescine content in transgenic plants was two-fold greater than that in WT plants. ‘FW’ refers to fresh leaf weight. ‘WT’ refers to non-transgenic control plants of wild type cotton. **: P<0.01.

An analysis of polyamine content demonstrated that the putrescine content in the four transgenic lines was, on average, two-fold greater than that in the control plants, (Fig 8).However, no significant differences in spermidine or spermine content were observed between the transgenic lines and the control plants.

### Analysis of the nitrogen content per unit weight in the leaves of transgenic cotton grown in the field

**Fig 9:**
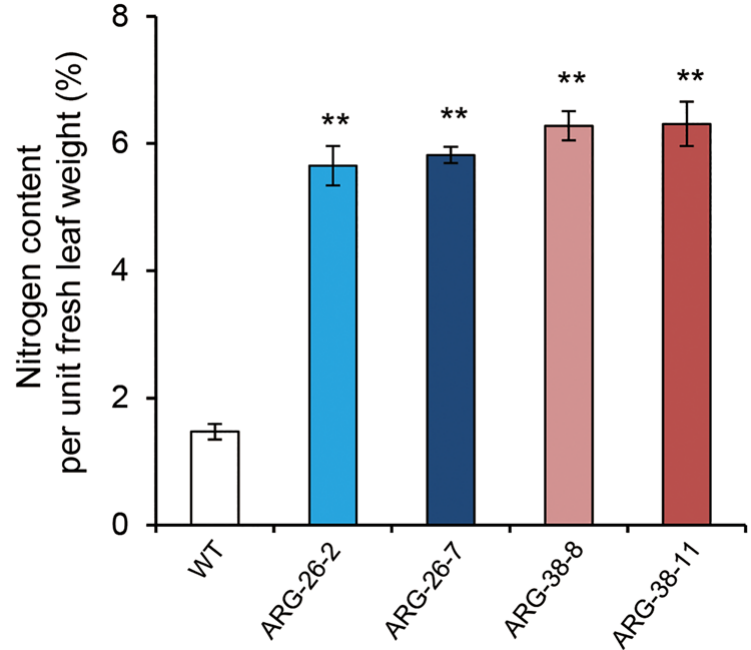
Comparisons of the nitrogen content per unit leaf weight in transgenic cotton compared with non-transgenic control plants under field conditions. The nitrogen content per unit leaf weight in ARG-26-2, ARG-26-7, ARG-38-8, and ARG-38-11were significantly increased compared with the WT. ‘WT’ refers to non-transgenic control plants of wild type cotton. **: P<0.01.

The nitrogen content per unit leaf weight is the typical empirical factor used to determine nitrogen use efficiency in plants. To detect whether the nitrogen content was altered, penultimate leaves from ARG-26-2, ARG-26-7, ARG-38-8, and ARG-38-11 plants were assayed to evaluate the amino acid and polyamine content and to measure the nitrogen content per unit leaf weight in cotton. The results demonstrated that *OsARG* expression significantly enhanced the accumulation of nitrogen in cotton leaves (Fig 9). The nitrogen content per unit leaf weight in transgenic plants was twofold greater than that in control plants (1.47%). The average nitrogen content values per unit leaf weight were 5.65%, 5.82%, 6.28%, and 6.31% in ARG-26-2, ARG-26-7, ARG-38-8, and ARG-38-11, respectively.

### Fiber length analysis of transgenic cotton grown in the field

**Fig 10:**
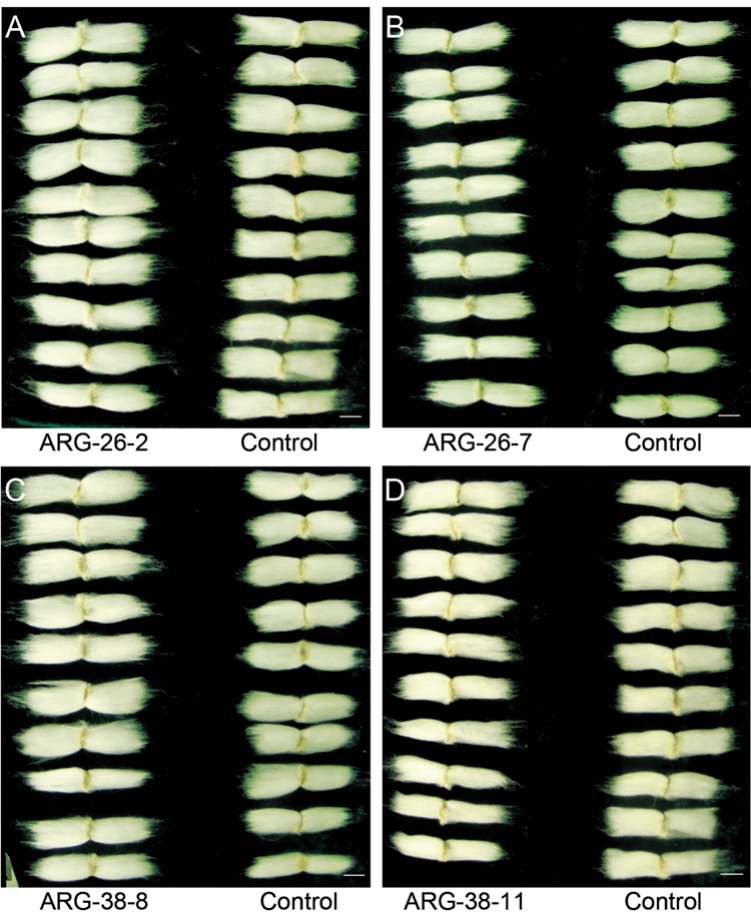
Fiber lengths of transgenic cotton are compared with Control. (A) the fiber length of ARG-26-2 compared with that of the Control. (B) the fiber length of ARG-26-7 compared with that of the Control. (C) the fiber length of ARG-38-8 compared with that of the Control. (D) the fiber length of ARG-38-11 compared with that of the Control. ‘Control’ refers to fiber of non-transgenic wild type plants. Scale bar, 5mm.

To detect whether *OsARG* expression affected cotton fiber length (an important agronomic trait), 30 independent cotton plants from each line were randomly selected in the field and used as samples for the measurement of fiber length. Transgenic fibers were longer than control fibers (Fig 10). The average fiber length in control plants was 29.36 mm, while the average cotton fiber lengths in ARG-26-2, ARG-26-7, ARG-38-8 and ARG-38-11were 31.94 mm, 32.00 mm, 32.68 mm, and 32.84 mm, respectively; note that these values were approximately two millimeters longer than the fibers of the control plants (Tables 2 and S2).

**Table 2:** Fiber length analysis in transgenic cotton and control plants.

	Cotton fiber length (mm)				
Number	Control	ARG-26-2	ARG-26-7	ARG-38-8	ARG-38-11
1	30.50	32.00	31.50	32.50	32.00
2	29.50	31.00	31.50	33.00	32.50
3	29.25	32.00	31.00	33.00	33.50
4	30.00	30.00	31.50	32.00	33.00
5	29.75	33.50	32.00	33.00	32.00
6	30.25	32.00	32.00	32.00	32.60
7	30.00	32.00	31.00	32.00	33.00
8	30.00	32.00	31.50	32.00	32.00
9	29.00	31.50	31.50	32.00	31.50
10	30.00	32.00	31.50	32.00	33.50
11	29.00	32.00	31.50	33.00	33.00
12	28.50	31.50	32.00	33.00	33.00
13	30.00	32.00	32.00	32.00	32.00
14	29.50	31.00	33.00	33.00	33.50
15	29.50	32.40	32.00	32.00	32.00
16	30.50	31.50	32.00	32.50	32.50
17	29.00	32.00	32.50	32.50	33.50
18	28.50	32.00	32.00	33.00	33.50
19	29.50	33.00	33.00	32.50	33.00
20	30.50	32.00	32.00	33.00	33.50
21	28.50	32.00	32.50	33.50	33.00
22	28.50	32.00	32.00	33.50	32.00
23	29.50	32.50	32.50	33.00	33.50
24	28.00	33.00	33.00	33.00	33.50
25	29.00	32.50	33.00	32.50	33.00
26	29.50	32.10	32.00	32.20	33.00
27	28.00	32.40	32.00	33.00	33.00
28	29.00	31.20	31.50	33.20	33.00
29	28.50	32.00	32.50	33.00	33.50
30	29.50	31.00	32.00	33.50	32.50
Average	29.36(±0.59)	31.94(±0.45)	32.00(±0.40)	32.68(±0.45)	32.84(±0.50)

ARG-26-2 and ARG-26-7 were bred from the ARG-26 line. ARG-38-8 and ARG-38-11 were bred from theARG-38 line. ‘Control’ refers to non-transgenic wild type plants.

## Discussion

In this study, we used the *AtALDH* mitochondrial transit peptide coding sequence to replace the *OsARG* mitochondrial transit peptide coding sequence and constructed an *OsARG* gene expression vector. Two *OsARG*-transformed cotton lines, ARG-26 and ARG-38, were generated via *Agrobacterium*-mediated genetic transformation. Transgenic cotton lines from the T3 generation were bred from ARG-26 and ARG-38. Southern blot analyses indicated that two copies and one copy of the *OsARG* gene were integrated into the ARG-26 and the ARG-38 genomes, respectively. Quantitative PCR analyses indicated that *OsARG* was highly transcribed in transgenic cotton plants. Enzyme activity analyses indicated that the enzyme activity of arginase in the transgenic cotton plants was significantly higher than that in control plants. These results confirmed that the rice arginase gene, *OsARG*, was successfully expressed and translated in the transgenic cotton lines.

### *OsARG* expression affects the morphology and development of the entire cotton seedling

During seed germination and seedling development, plant nitrogen nutrition is mainly acquired from the metabolism of storage Arg in cotyledons. At the early stages of seedling development, 90% of soluble nitrogen is obtained from Arg [6, 7]. The products of Arg metabolism can be used in the synthesis of polyamines, amino acids, proteins, and NO [11, 12]. These synthesized molecules are involved in the regulation of plant growth and development, as well as in establishing plant architecture; they also have roles in various components of the photosynthetic machinery [13, 16, 25, 26]. In this study, we cultured transgenic cotton seedlings on different medias with varying nitrogen content. The results demonstrated that seedling height and taproot length in the transgenic plants differed from the control (S1 Fig). When transgenic seedlings were grown on 1/2MS medium lacking NH_4_NO_3_ and KNO_3_, taproot elongation was enhanced, and seedling height and lateral root development were inhibited. But when transgenic seedlings were grown on 1/2MS medium that contained a four-fold increase in NH_4_NO_3_content, the phenotypes of the transgenic seedlings for height, taproot length, and average lateral root length were recovered to the levels seen with the 1/2 MS medium treatment. We speculate that increased arginase enzyme activity resulted in speed up usage of stores of internal nitrogen and external nitrogen (from the medium), this could have caused alterations in the seedling height and taproot length.

Nitric oxide synthase (NOS) uses Arg as a substrate to produce nitric oxide (NO). It was reported that the NOS activity and the NO content increased in *Arabidopsis thaliana* Arginase-negative mutation plants. It is known that NOS activity can be inhibited by increased argniase activity [13]. Increases in NO content on medium can affect the development of plant roots; this has been reported in roots exhibiting gravitropic bending (*Glycine max*) [27], in the promotion of lateral root development (*Solanum lycopersicum*)[28], the development of root hairs (*Arabidopsis thaliana*)[29], and adventitious roots development (*Cucumissativus*)[30]. In this study, as compared with the control plants, lateral root development in ARG-26 and ARG-38 seedlings was inhibited when grown on 1/2 MS medium with varying nitrogen content. The number of lateral roots was significantly decreased in the transgenic plants as compared to control plants. The average length of the lateral roots of ARG-26 and ARG-38 seedlings decreased when plants were grown on 1/2 MS medium and 1/2 MS medium without NH_4_NO_3_ and KNO_3_. The roots NO content analysis results showed that using Arg to synthesize NO was the main source of NO in cotton roots, and this metabolism process could be inhibited by increased arginase enzyme activity. It is likely that expression of the rice arginase gene *OsARG* increased the total arginase activity in cotton and affected the development of roots by altering NO content in the roots.

### *OsARG* expression can increase the nitrogen content and fiber length in cotton grown under field conditions

Arg is one of the major amino acids involved in nitrogen recycling and remobilization in plant development processes. Arg metabolism is closely related with the development of reproductive organs. 70 to 90 percent of seed nitrogen is obtained from vegetative organs by amino acid transporting processes [31, 32]. In rice leaves, roots, and seeds, Arg accounts for 7.5 to 11.6 percent of total free amino acid content [33, 34, 35]. Arg nitrogen can be recycled by the coordinated action of arginase and urease in order to meet the metabolic demands of developing organs. This process plays a crucial role in rice nitrogen use efficiency [16].

During cotton development, the appearance of the first bud is an important turning point in the transition from the vegetative growth stage to the reproductive growth stage. The nitrogen content at this point will affect the development of flowers, bolls, and fibers during later phases of reproductive growth. Our results indicate that expression of the *OsARG* gene in cotton increases the nitrogen content in transgenic cotton leaves when the first bud appears in plants grown under field conditions. The content of polyamines and nitrogen increased by more than twofold in the transgenic plants as compared to that in control plants. These results indicate that arginase may be associated with cotton nitrogen transition. We speculate that expression of the *OsARG* gene in cotton has a positive influence on cotton nitrogen use efficiency.

Cotton fibers develop from a single epidermal cell of cotton seeds, and the quality of fiber is closely related with the seed development process [1]. In our previous studies, we found that cotton arginase was strongly expressed during early-stage fiber development (data not shown). Compared with the controls, the fiber lengths of *OsARG*-expressing cotton plants was increased about by 2mm. These results suggested that rice arginase gene, *OsARG*, can effectively execute its function in transgenic upland cotton to increase nitrogen transit efficiency and may be associate with fiber development. In addition, the *OsARG* gene can be used as a potential gene for cotton improvement efforts aimed at improving both nitrogen use efficiency and fiber length in cotton breeding programs.

## Supporting Information

S1 Fig Morphology of transgenic cotton seedlings grown on 1/2 strength MS media containing varying nitrogen content. (DOCX)

S1 Table Primers used in this study. (DOCX)

S2 Table Average fiber length of transgenic cotton in different years (DOCX)
