# Supplementary material for: Expression of the Rice Arginase Gene OsARG in Cotton Influences the Morphology and Nitrogen Transition of Seedlings
Source: PLoS One. 2015 Nov 3;10(11):e0141530. doi: 10.1371/journal.pone.0141530 (PMC4631492; doi:10.1371/journal.pone.0141530)

**Supplementary Figure legends**

**S1 Fig. Morphology of transgenic cotton seedlings grown on various 1/2 MS media.**

A, the seedlings were grown on 1/2 MS medium.

B, the seedlings were grown on 1/2 MS medium lacking NH_4_NO_3_ and KNO_3_.

C, the seedlings were grown on 1/2 MS medium with 4×NH_4_NO_3_.

Scale bar, 1cm.

**S1 Fig**


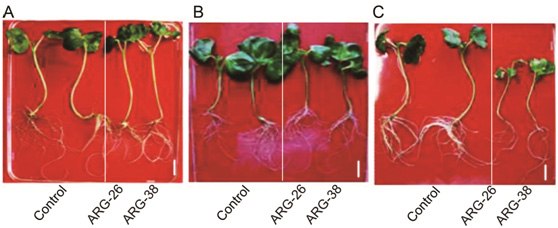

Supplement: S1 Fig — (DOCX) [file pone.0141530.s001.docx]
