# Supplementary material for: Expression of the Rice Arginase Gene OsARG in Cotton Influences the Morphology and Nitrogen Transition of Seedlings
Source: PLoS One. 2015 Nov 3;10(11):e0141530. doi: 10.1371/journal.pone.0141530 (PMC4631492; doi:10.1371/journal.pone.0141530)
Supplement: S1 Table — (DOCX) [file pone.0141530.s002.docx]

**S1Table Primers used in this study**

| Primer name | Sequences |
| --- | --- |
| ARG_FP | CTGCAG***ATGGCATCAAGAAGAGTTTCTTCGCTGCTCTCTCGCTCTTTCATGTCCTCCTCACGTTCTATC***GCCGCCAAGGTGTCGGCGG |
| ARG_RP | CTCGAGTCACTTGGAGATCTTGGCTGTGAG |
| *OsARG*-F | GCCGCCAAGGTGTCGGCGG |
| *OsARG*-R | TCACTTGGAGATCTTGGCTGTGAG |
| *EF1α*_F | AGACCACCAAGTACTACTGCAC |
| *EF1α*_R | CCACCAATCTTGTACACATCC |
| *OsARG*_QPCR_FP | GATAGGGAGAAGCTCGAAAG |
| *OsARG*_QPCR_RP | TCCTGGCTCAATGTGAGAGAC |

Underlined letters in the ARG_FP and ARG_RP primers represent restriction endonuclease recognition sites for *Pst*I and *Xho*I. Letters that are in bold and italicized represent the mitochondrial transit peptide coding sequence. The remaining letters in the ARG_FP and ARG_RP primers correspond to the *OsARG* CDS sequence.
