# Supplementary material for: Expression of the Rice Arginase Gene OsARG in Cotton Influences the Morphology and Nitrogen Transition of Seedlings
Source: PLoS One. 2015 Nov 3;10(11):e0141530. doi: 10.1371/journal.pone.0141530 (PMC4631492; doi:10.1371/journal.pone.0141530)
Supplement: S2 Table — (DOCX) [file pone.0141530.s003.docx]

**S2 Table Average fiber length of transgenic cotton in different years**

| Cotton line | Average fiber length (mm) | | | |
| --- | --- | --- | --- | --- |
|  | 2011 | 2012 | 2013 | 2014 |
| Non-transgenic | 31.31±0.72 (30) | 30.8±0.46 (30) | 30.54±0.33 (30) | 29.36±0.59 (30) |
| ARG-26 | 34.28 ±0.02 (1) | 33.94 ±0.78 (15) | 34.01±0.83 (26) | 31.97±0.42 (30) |
| ARG-38 | 33.81 ±0.01 (1) | 33.45±0.92 (26) | 33.95±0.76 (28) | 32.76±0.49 (30) |

Figures in brackets refer to number of individual plants that were sampled to evaluate fiber length.
